# The Medical Aspects of the Divorce Question

**Published:** 1912-11-30

**Authors:** 


### THE MEDICAL ASPECTS OF THE DIVORCE QUESTION.

The recently issued Reports of the Royal Commission on Divorce will doubtless be exposed to much criticism both from experts and from those who take merely a sentimental, and therefore, perhaps, prejudiced, interest in the matters with which they deal. The main conclusions embodied in the two Reports will by this time be well known to our readers. Briefly stated, the opinion of the Majority of the Commissioners is that greater facilities for divorce should be provided in order to meet those cases of undoubted hardship and injustice occasioned by the present inequality before the law of the two sexes in respect to this important matter. That of the Minority, on the other hand, is that nothing should be done to make divorce more easy than it already is. The signatories to the Minority Report do not deny that there are cases of hardship and injustice, but they base, apparently, their whole argument on the premiss that such hardships must inevitably occur (they are not rare), and that to obviate them by introducing new facilities and untried methods of assuagement will be to open the door to possible abuses the influence of which is likely far to exceed in gravity the evils occasioned by the existing system.

It is not with these two points of view that the medico-sociologist, who, of all persons in the community should be the most interested in the question of divorce, is likely to enter into debate. The divergences between them are so great that it presupposes a concession of principle before one side can give up its claim to stand on a securely logical footing. Most of us will be content with the admitted fact that the present system presses unequally, and therefore unjustly, upon the two sexes. No one who has studied the question of divorce and marriage restriction can for a moment doubt that such inequality is, in the present stage of our civilisation, uncalled for and obnoxious; it serves no useful purpose now, whatever it may have done in the past. We are, therefore, gratified to find that the consensus among the Commissioners is distinctly in favour of abolishing sex inequality. It is equally gratifying to find that there exists a similar agreement with regard to facilitating the dissolution of the legal union between poor people who cannot afford to pay the heavy law fees demanded in ordinary cases, and who can scarcely avail themselves of the circumlocutory and dilatory machinery provided for those who desire to sue *in forma pauperis*. Medical men, who have as much experience, perhaps, as lawyers of the hardships endured by parties to an ill-matched marriage, know how difficult is the position of the poor husband or the poor wife saddled with a partner whose partnership means a negation of all the ordinarily accepted interpretations of conjugal felicity. But medical men know also how extremely difficult it is to define the limits within which these ill assortments may be permitted to continue without detriment to the offspring. For that reason they will scrutinise very closely the recommendations made by the Commissioners, especially those embodied in the Report of the Majority. For we take it that this Report will form the basis of whatever amending legislation is to be introduced next year. The present administration at the helm appears to be unduly favourable to new suggestions for modifying society, and not altogether indisposed to accept the views of extremists whose ideals are not consonant with what we have thought right in the past. It is, therefore, quite within the bounds of possibility that unless the public bestirs itself to take an active interest in subjects which, after all, touch it as closely as do Home Rule and Tariff Reform and the Disestablishment of the Church in Wales, we shall next year find a Bill which tries to enact in statutory form the drastic recommendations of the Majority Report. One of the most serious of these undoubtedly is the suggestion that "incurable insanity" in one partner, after several years' duration, shall be deemed sufficient ground for divorce. This, we venture to think, is a recommendation that should be submitted to the closest scrutiny. As it stands the recommendation is altogether too wide or too narrow. The substitution of incurable disease for the terms incurable insanity will make it at least a logical suggestion, for we fail to see the difference between a union wherein one partner is afflicted with, say, general paralysis, and one in which husband or wife suffers from locomotor ataxia. The difference, if there is one, is one of degree, and to the scientific as well as to the sentimental, the degree is a varying one, sometimes so small as to be inappreciable. Chronic inebriety, lifelong imprisonment, and temporary punishment for gross sexual crimes appear to us to be much more suitable and legitimate grounds for dissolving the marriage than what is somewhat vaguely styled "incurable insanity." It behoves the medical profession to interest itself in the matter and to make the public acquainted with the scientific and medical aspects of the problem. Only by doing so can the profession acquit itself of what is a stern public duty, and counter the wild suggestions of those who wish to regenerate the race at the expense of a section whose interests for the moment seem overshadowed by those of the community at large.

